# Fano-Resonant Hybrid Metamaterial for Enhanced Nonlinear Tunability and Hysteresis Behavior

**DOI:** 10.34133/2021/9754083

**Published:** 2021-08-13

**Authors:** Yuancheng Fan, Xuan He, Fuli Zhang, Weiqi Cai, Chang Li, Quanhong Fu, Nataliia V. Sydorchuk, Sergey L. Prosvirnin

**Affiliations:** ^1^Key Laboratory of Light Field Manipulation and Information Acquisition, Ministry of Industry and Information Technology and School of Physical Science and Technology, Northwestern Polytechnical University, Xi'an 710129, China; ^2^Research & Development Institute of Northwestern Polytechnical University in Shenzhen, Shenzhen 518057, China; ^3^Institute of Radio Astronomy, National Academy of Sciences of Ukraine, Kharkiv 61002, Ukraine; ^4^Karazin Kharkiv National University, Kharkiv 61077, Ukraine

## Abstract

Artificial resonant metamaterial with subwavelength localized filed is promising for advanced nonlinear photonic applications. In this article, we demonstrate enhanced nonlinear frequency-agile response and hysteresis tunability in a Fano-resonant hybrid metamaterial. A ceramic cuboid is electromagnetically coupled with metal cut-wire structure to excite the high-Q Fano-resonant mode in the dielectric/metal hybrid metamaterial. It is found that the significant nonlinear response of the ceramic cuboid can be employed for realization of tunable metamaterials by exciting its magnetic mode, and the trapped mode with an asymmetric Fano-like resonance is beneficial to achieve notable nonlinear modulation on the scattering spectrum. The nonlinear tunability of both the ceramic structure and the ceramic/metal hybrid metamaterial is promising to extend the operation band of metamaterials, providing possibility in practical applications with enhanced light-matter interactions.

## 1. Introduction

Beginning of metamaterial refers to a class of artificial structures that possess properties unattainable in nature materials. These structures usually consist of subwavelength-sized metallic particles (known as meta-atoms) [1–4]. In specific conditions when the real part of the permittivity of metal is negative, these structures are named also plasmonic metamaterials. Recently, a type of resonance with sharp asymmetric line-shape, namely, Fano resonance [5–7] attracted an increasing amount of interest in the research field of plasmonic metamaterials [8, 9]. The Fano resonance originates from coherent interference between a narrow discrete mode and a board mode spectrum. It has been explored in various subwavelength structures made from metals including side-coupled waveguide-cavity systems [10], gold gratings [11, 12], meta-atoms or plasmonic particles with structural asymmetry [13–15], and gold nanoparticles/nanoshell heptamers [16, 17]. Fano resonance is interesting for applications in ultrasensitive sensing [18] and filtering [19], owing to sharp changing of its electromagnetic properties within the resonant regime [6, 20]. The significant enhancement of the electromagnetic field intensity and novel field localization is associated with Fano resonance in the open planar structures [21, 22], and as consequence, the enhanced group delay [23] can be utilized to essentially boost light-matter interactions.

Mie resonance of dielectric particle is an alternative route for the realization of subwavelength meta-atoms. In 2002, O'Brien and Pendry [24] developed the effective medium description of high permittivity dielectric rods and found that the local resonance (single-scatter resonance) of the rod shows a negative magnetic permeability at microwave frequencies. Holloway et al. [25] found that the effective permeability and permittivity of a matrix with embedded magneto-dielectric spherical particles can be simultaneously negative. Zhao et al. [26] demonstrated negative permeability at microwave frequencies in a three-dimensional all-dielectric composite consisting of an array of dielectric cubes [BaSrTiO_3_ (BST)] on a teflon substrate. Peng et al. [27] experimentally observed a left-handed behavior in a prism of periodically/randomly arranged BST rods. Compact dielectric particles were manifested as a building block for low-loss magnetic metamaterials [28]. Various all-dielectric metamaterials have been demonstrated in a wide range from microwave to optical frequencies [29–33]. Compared to metallic-based plasmonic metamaterials with Joule losses, the structures operating on Mie resonances of dielectric particles are promising for low-loss metamaterials [29–31, 34–45].

Recently, all-dielectric metamaterials, e.g., dielectric oligomers and a dielectric particle combined with a metal wire, have been proposed to produce a Fano resonance [46–49]. It is well known that the steep dispersion of the Fano resonance is promising for enhancing nonlinear effects. Furthermore, it is noteworthy that some dielectric materials incorporating in conjunction with metallic metamaterials are helpful for achieving active control of light propagation in, e.g., terahertz regime [50–52] or even nonlinear tunable metamaterials [45, 53–56]. In this paper, we demonstrate that the nonlinear properties of the ceramic cuboid inclusion in a dielectric/metal composite, or hybrid metamaterial, can be employed for dynamically tuning the Fano resonance. It is found that the Fano resonant is induced by coupling the dipolar mode of a cut-wire with a ceramic structure. And then the strongly localized field of a Fano-resonant mode takes effect in enhancing the nonlinear property of the dielectric ceramic cuboid. The nonlinear tunability of the Fano resonance and hysteresis/bistable response is theoretically and experimentally demonstrated in the hybrid metamaterial. The enhanced nonlinear response in Fano-resonant metamaterial may be extended for low-power optical nonlinear and related applications taking advantages of nonlinear nanophotonics.

## 2. Results and Discussion

**Figure 1:**
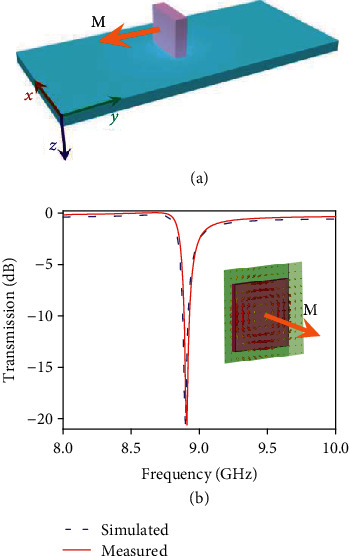
Linear response of the dielectric metamaterial made of ceramic: (a) schematic of a ceramic cuboid placed at the center of substrate crossed a X-band waveguide; (b) calculated and measured transmission spectra for the ceramic metamaterial, the inset shows the electric field profile on the cross-section of the cuboid dielectric resonator at the resonant frequency.

A simplest way to experimentally study of electromagnetic properties of a metamaterial is to get its waveguide model characteristics. The metamaterial and the measurement setup are schematically illustrated in Figure 1(a). A standard X-band waveguide was employed for the study of dielectric/metal hybrid metamaterials. The metamaterial sample was placed on the dielectric substrate in the cross-section of the waveguide at *xy*-plane. The substrate thickness is 1.0 mm. Its relative permittivity is 2.1 with a negligible imaginary part.

First, we studied the resonant response of a single ceramic cuboid with dimensions of 3.5 mm × 3.5 mm × 1.0 mm fabricated from calcium titanate (CaTiO_3_) doped by 1 wt.% ZrO_2_ which is a low-loss material with a high relative permittivity value ranged up to approximately 120. The ceramic cuboid was placed by its narrow rectangular cross-section along *x*-direction to excite its first-order Mie resonance within the working band of the waveguide. The calculated transmission spectrum of the single ceramic cuboid is shown in Figure 1(b) (dashed violet curve), where we can see a sharp resonance near frequency of 8.9 GHz. A Finite-Difference-Time-Domain (FDTD) software package was employed for full-wave simulations. To get a better understanding of the resonant mechanism, we show the distribution of the electric field along the cross-section of the cuboid ceramic resonator at the resonant frequency in the inset of Figure 1(b). The resonant mode of the ceramic cuboid is the nearly same as the TE_01_ mode of a spherical resonator [27–31]. The resonance originates from the excitation of the magnetic dipole of the cuboid. This effective magnetic dipole is directed along *y*-axis. It can be seen that the fields are mostly localized in the subwavelength ceramic cuboid, which intuitively would be helpful in the enhancement of nonlinear polarization ability of material as that in metallic metamaterials [57, 58]. In the experiment, we fixed the ceramic cuboid in the same manner as in the simulations. The transmission through the sample was measured with a vector network analyzer (VNA) in the range of 8 ∼ 12 GHz, and the measured result agrees well with the calculated transmission spectrum, as shown in Figure 1(b).

We then irradiated the sample with an electromagnetic wave of different intensity to characterize the nonlinear properties of the ceramic cuboid. The transmission spectra were measured at different levels of input power from 15 dBm to 30 dBm.

**Figure 2:**
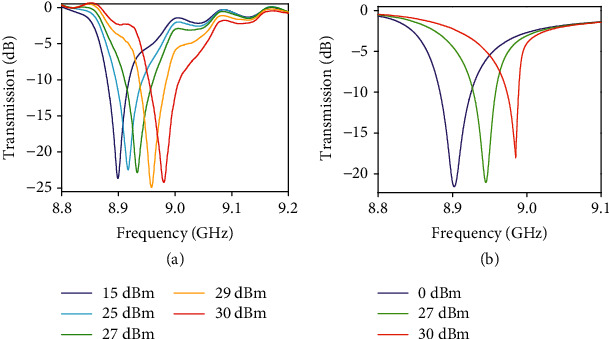
(a) Measured transmission spectra with different input power levels from 15 to 30 dBm. (b) Simulated transmission spectra with different input power levels. The cuboid is placed at a center of the waveguide rectangular section on a substrate and fixed by a narrow facet on the substrate surface (see Figure 1(a)).

The measured transmission spectra around the magnetic dipole resonant frequency are plotted in Figure 2(a) for input power of 15 dBm, 23 dBm, 25 dBm, 27 dBm, 29 dBm, and 30 dBm, where the nonlinear frequency shift of the resonance becomes notable. For low input powers, such as 15 dBm and below, the measured resonant frequency of the magnetic mode is 8.9 GHz (see the violet curve in Figure 2(a)), which is the same as the linear response in Figure 1(b). The magnetic resonance starts to shift to higher frequencies when the power is higher than 15 dBm, and the shift becomes bigger as the power further increases beyond 25 dBm. The nonlinear shift about 100 MHz in the range from 15 dBm to 30 dBm should be described by higher orders of electric polarizability of the cuboid material. The nonlinear shift of the resonance originates from the heating effect in the resonant ceramic cuboid associated with the strong field localization and nonlinear susceptibilities of ceramics; as a result, a blue shift with increasing the environmental temperature takes place [56].

Thus, in the nonlinear regime, the incident electromagnetic field intensity may be large enough to produce heating inside the lossy resonant metamaterial that can essentially modify the permittivity of the ceramic cuboid. Because heating is in dependence on energy of the electromagnetic field dissipated in the cuboid, it is proportional to an inner electric field strength squared. Consequently, the effect could be interpreted as a third-order Kerr nonlinearity [59] with the following expression of relative permittivity of ceramic

(1) $$\begin{array}{c} \varepsilon =\varepsilon^{\prime }-2i\left(\frac{\sigma }{f}\right){\text{c}}^{2}10^{-7}-\chi {\left|E\right|}^{2}, \end{array}$$

where *ε*′ is a real part of relative permittivity of cuboid ceramics at linear regime, *σ* is a ceramics conductivity, *f* is frequency, *c* is a speed of light in free space, and *χ* is a coefficient of Kerr nonlinearity. As a result, the reflection and transmission of metamaterial depend on the incident wave amplitude and may be controllable.

A FEM-based electromagnetic solver was employed for full-wave simulations to study metamaterial properties in the nonlinear regime. First of all, a nonlinear response simulation was made for waveguide samples of metamaterials consisted of a ceramic cuboid placed on a substrate. As a result of comparison with measured response, we have estimated that the ceramic material of the cuboid is characterized by relative permittivity of *ε*′ = 122.7 at small electric field intensity corresponding to the linear regime of polarizability approximation and by electrical conductivity *σ* = 0.062 S/m. Also, we have defined a nonlinear property of the cuboid dielectric as Kerr nonlinearity with coefficient *χ* = 0.45 · 10^−8^ m^2^/V^2^ at |*E*|^2^. A comparison of measurement (Figure 2(a)) and simulation (Figure 2(b)) results shows a good correspondence. All results of simulations presented below have been found by using these constitutive parameters. Discontinuities of some dependencies correspond to resonant frequency ranges with a bistable response of the structure on excitation by the intensive electromagnetic wave.

**Figure 3:**
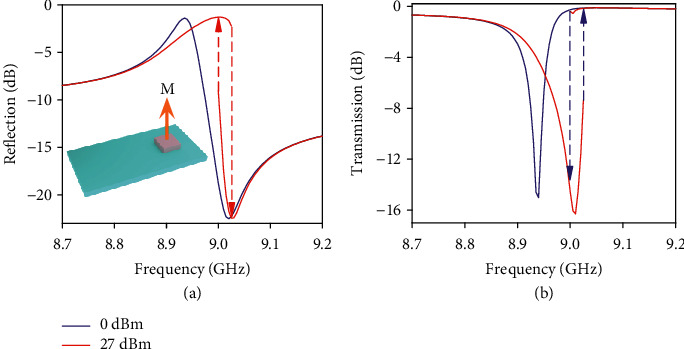
Simulated reflection (a) and transmission (b) spectra at two different input power level (0 dBm and 27 dBm, correspondingly violet and red curves) near the resonant frequency of effective magnetic dipole of the ceramic cuboid. The cuboid is placed with a shift from a center of the waveguide rectangular section and fixed on the substrate by a wide facet (see inserted sketch).

**Figure 4:**
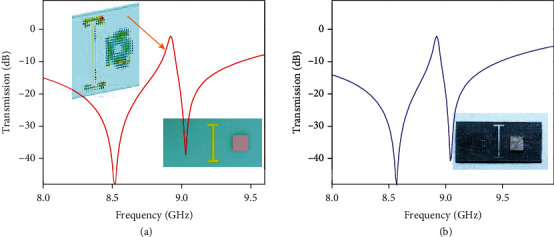
Schematic of the ceramic/metal hybrid metamaterial and the linear response of the metamaterial. (a) Calculated and (b) measured transmission spectra of the ceramic/metal hybrid metamaterial. Right inset in (a): an illustration diagram of the ceramic/metal hybrid metamaterial comprised of I-shaped metal pattern placed at the center of the cross-section of waveguide, and a ceramic cuboid fixed at 4 mm away from the I-shaped film in *y*-axis direction. Left inset in (a): electric field distribution of the sharp trapped mode (at 8.9 GHz). Inset in (b): a photograph of the sample measured in experiment.

The asymmetric line-shape of the Fano-resonance [6–9] was theoretically explained as the superposition of discrete and continuum transitions in quantum mechanics [8]. It is attractive for applications in various systems, especially in nanostructures and plasmonic metamaterials [6, 9, 10, 21] which have exotic performance in sensitive sensors, switching, laser technique, and nonlinear optics in recent years. The Fano resonance is interesting for the intrinsic sharp changing on the spectrum between the spectral dip and peak, which can be used to obtain spectrum modulation under tiny changing of the surrounding medium. A typical Fano-kind resonance may be observed by putting the same cuboid resonator on the substrate by its wide facet but asymmetrically relative to the waveguide cross-section center. We have positioned the cuboid with a 4 mm shift in *y*-direction from a central point. The shift is employed to excite a resonant field inside the cuboid that would be equivalent to the *z*-directed magnetic dipole. Spectral characteristics of the metamaterial are presented in Figures 3(a) and 3(b). It is clear that there exists nonlinear shift of the resonant frequency, and the high-frequency side of the resonance becomes sharper at an input level of 27 dBm which is the characteristic of nonlinear hysteresis. We further introduced the studied ceramic cuboid into a metallic metamaterial (see Figure 4) for nonlinear tuning the Fano line-shape response of the ceramic/metal hybrid metamaterial for remarkable modulation of the transmission of electromagnetic waves.

The ceramic/metal hybrid metamaterial is shown in Figure 4(a), an I-shaped metal film is patterned at the center of a teflon substrate (with the same size as the cross-section of a waveguide), and the ceramic cuboid is fixed 4 mm away from the I-particle in *y*-axis direction. The I-shaped metamaterial has been intensively studied for a broad electric response, which will play the role of background continuum in our classical studied Fano-resonant structure. The electric resonance of the I-shaped metamaterial is around 8.9 GHz. The broad resonance covers the frequency band 8 ∼ 10 GHz we are concerning with in this study. The sharp magnetic resonance of the ceramic cuboid occurs in the resonant regime of the electric mode, then, the closely spaced ceramic resonator and the metal resonator will couple with each other under external stimuli [60, 61] and together show quantum-like superposition on the scattering spectrum. In Figure 4(a), we plot the calculated spectrum of transmission through the ceramic/metal hybrid metamaterial. A sharp asymmetric resonance can be seen near 8.9 GHz, and it is exactly the magnetic resonance of the single ceramic cuboid. The electric field distribution of the sharp trapped mode is also presented as the left inset in Figure 4(a), from which we can clearly see that the fundamental magnetic mode of the ceramic cuboid (with electric fields around the *z*-axis of the cuboid) is well excited. The excitation is due to the overlapping of the magnetic component of the magnetic mode with the magnetic field around the metal resonator. Photograph of the fabricated ceramic/metal hybrid metamaterial sample is shown in Figure 4(b), and the measured linear transmission spectrum is the plot in Figure 4(b). The measured linear transmission agrees well with the calculated transmission spectrum with only slight resonance shift that is common existing in experiments.

**Figure 5:**
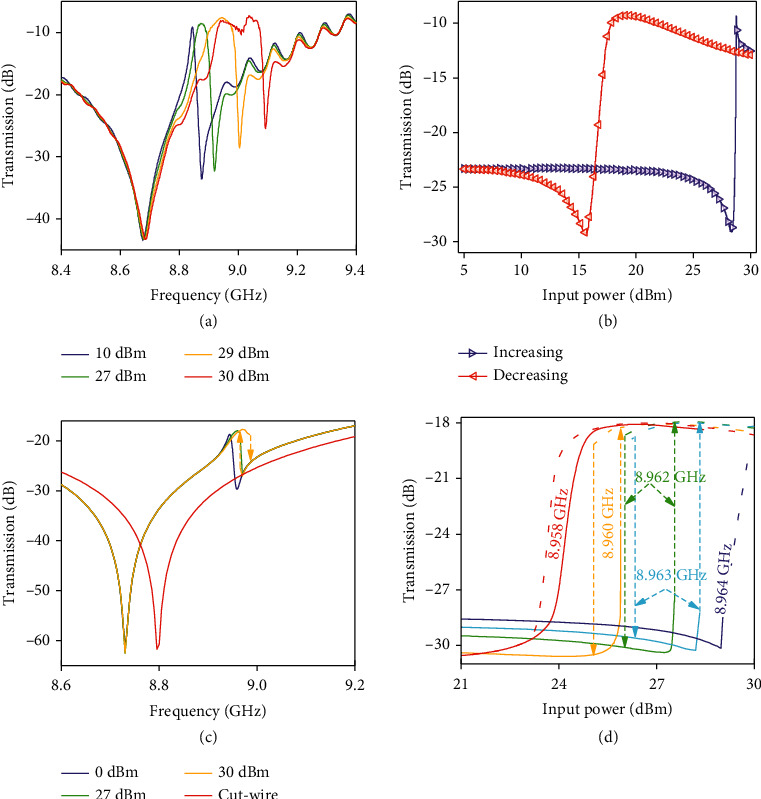
Measured and theoretically calculated nonlinear tuning and hysteresis behavior of the hybrid metamaterial. (a) Measured transmission spectra at different levels of input power near the Fano-like resonance frequency for the ceramic/metal hybrid metamaterial. (b) Measured transmission (in dB) of hybrid metamaterial in the frequency of 8.963 GHz as a function of input power in the range of 6 ∼ 30 dBm: right triangles and left triangles are for forward and backward directions in bidirectional sweep in input power. (c) Simulated spectra of transmission at different input power levels near the Fano-like resonance frequency for the ceramic/metal hybrid metamaterial and the wire-cut structure (red curve). (d) Simulated transmission (in dB) of hybrid metamaterial at frequencies 8.958, 8.960, 8.962, and 8.963 GHz as a function of input power for scenarios of increasing (solid lines) and decreasing (dash lines) input power.

By incorporating the ceramic cuboid in the I-shaped metallic metamaterial with a low-Q broad resonance, a sharp Fano resonance can be achieved. The sharp Fano resonance shows a rapid increase in the group delay [48], which is essential in the enhancement of light-matter interactions. The transmission of the ceramic/metal hybrid metamaterial is then measured to characterize its nonlinear properties. The transmission spectra are measured at different input power levels from 5 dBm to 30 dBm. The measured transmission spectra are plotted in Figure 5(a) for intensities of 10 dBm, 27 dBm, 29 dBm, and 30 dBm, where the nonlinear frequency shift of the Fano resonance becomes notable. The Fano-resonance starts to shift to the higher frequency when the power is higher than 20 dBm, and the shift becomes fast as the power further increasing beyond 27 dBm, a more than 200 MHz shift of the Fano-resonant dip was achieved when the input power reaches up to 30 dBm, and the nonlinear shift is associated with notable modulation on the transmission (a more than 25 dB).

Furthermore, we notice that the nonlinear modulation on the transmission is neglectable outside the Fano resonance, confirming again the Fano resonance is induced by the sharp magnetic resonance of the ceramic cuboid, which is essential in enhancing nonlinear interactions between electromagnetic waves and our ceramic/metal hybrid metamaterial.

Since that, Fano resonance of the hybrid metamaterial can significantly enhance the nonlinear response of the ceramic cuboid through the localized electromagnetic field in the subwavelength cuboid and the enhanced group delay. It is interesting to study the dynamical feedback, or hysteresis/bistable behavior, of the resonant hybrid metamaterial. Bistability is promising in the application of all-optical logic [58]. We controlled the intensity of a monochromatic input signal within the range 6 ∼ 30 dBm to demonstrate the bistable response of the hybrid system.  Figure 5(b) presents the transmission intensity at 8.963 GHz with respect to bidirectional sweep (right-triangle marker line: increasing, left-triangle marker line: decreasing) of the input power. It can be seen that the transmission has different values for the bidirectional sweep, a modulation on transmission up to 15 dB can be achieved in the range 17 ∼ 28 dBm. These two power levels define a regime of input power to trigger the two-valued transmission at the same frequency, which might be used for a nonlinear switch or other logic devices [62].

We deal with two physically different resonant particles which are constitutive parts of planar metamaterials. The first one is subwavelength ceramic cuboid with a high value of permittivity. Its lowest resonant frequency corresponds to electromagnetic field distribution of an equivalent magnetic dipole. In resonance, a magnetic dipole moment *M* of the cuboid is directed orthogonal to its wide facets (see Figure 1). The cuboid resonant field is excited by an electric field of basic waveguide mode. If the cuboid is fixed in waveguide in such a way that the vector *M* is directed parallel to *y*-axis of coordinate frame (as it is shown in Figure 1), boundary values related to the fields on waveguide walls make the resonant field very close to a classical trapped-mode field regime with a small coupling to waveguide modes propagated away of cuboid location. In fact, the value of coupling depends mainly from a size of cuboid in *z*-direction. The corresponding field configuration is different from a field of an usual size-resonance, and this difference appears as a larger quality factor and asymmetric Fano-like resonance spectrum.

A priori, it is known that the permittivity of ceramics used to produce the cuboid depends on the temperature. For measurement or simulation of response, we assume the temperature distribution has been set stable after some heating time at every level of the incident power. Thus, we can assume the permittivity of ceramics designed to any point of cuboid depends on electric field intensity in this point and has been defined by Kerr expression (1). The numerical solution of the self-consisting problem of determining field intensity distribution is solved. These data were used to calculate the transmission coefficient for metamaterial and the calculated transmission spectra are presented in Figure 2(b). At input power 30 dBm, we have observed a very sharp resonance with a shape of the spectral line near to a limit after which the spectral response of a bistable regime takes place.

We then put the cuboid on the substrate by its wide facet (see insert in Figure 3(a)). In this arrangement of the metamaterial structure, the value of quality factor is restricted only by dissipation losses because the radiation power losses may be decreased up to zero by placing the cuboid in a center of the waveguide cross-section. Our setup was made with the asymmetrically placed cuboid for good excitation of a resonance in the structure consisted of elements produced from common lossy media. The metamaterial manifests a typical spectral response related to the excitation of trapped mode resonance that has asymmetry Fano-liked shape (see Figures 3(a) and 3(b)). The input power level needed to observe such nonlinear effect as hysteresis of response is approximately twice lower in comparison with metamaterial consisted of cuboids mounted on narrow facets.

The second kind of particle used in our nonlinear metamaterial study is the cut-wire or I-shaped metallic dipole incorporated in the structure. The frequency dependence of the transmission coefficient of the alone I-shaped dipole placed on a substrate in the waveguide is shown in Figure 5(c). In the hybrid structure, the wire dipole produces some desired background of transmission which can be modified in the tuned resonance by using the ceramic nonlinear cuboid. The corresponding experimental and simulated results are presented in Figures 4, 5(a) and 5(c), respectively. The sharper resonances in both experimental and calculated spectra at increased input levels are corresponding to the nonlinear hysteresis. The hybrid metamaterial manifests hysteresis/bistable transmission as a response on increasing and decreasing input power at frequencies near to resonance frequency of the linear regime (see Figures 5(b) and 5(d)). It is obvious that the width of hysteresis loop is very sensitive to the frequency of input radiation which could be used for precise spectral sensing.

## 3. Conclusion

In summary, we have shown that a ceramic cuboid can be used itself or put into a hybrid metamaterial as a nonlinear inclusion to effectively induce and tune the Fano resonance of the dielectric/metal hybrid system. It is found that the significant nonlinear response of the ceramic cuboid can be employed for realizing tunable metamaterials by exciting its magnetic mode, and the trapped mode with an asymmetry Fano-like resonance is beneficial to achieve notable nonlinear modulation on the scattering spectrum. The nonlinear tunability of both the ceramic structure and the ceramic/metal hybrid metamaterial is promising to extend the working band of metamaterials, providing possibility in practical applications with enhanced light-matter interactions.

## 4. Materials and Methods

### 4.1. Sample Preparation

The printed circuit board (PCB) technique was used to manufacture the I-shape copper wire, and the structure was printed on 1 mm thick Teflon substrate. The relative permittivity equals 2.65 and loss tangent equals 0.001. The substrate was cut to a dimension of 22.86 × 10.16 mm^2^ corresponding to the cross-section of waveguide, simultaneously ensuring the copper wire at the center of the base. The dielectric cuboid fabricated from calcium titanate (CaTiO_3_) doped by 1 wt.% ZrO_2_ which is a low-loss material with a high relative permittivity value ranged up to approximately 120. The cuboid was sticking to the same surface of the base printed copper wire by double-sided adhesive.

### 4.2. Measurements

In the experiments, the sample was placed in a WR-90 rectangular waveguide and was measured by a vector network analyzer. The cuboid is placed at a distance away from center of substrate along the *y*-axis under the assist of microscope. For the nonlinear measurement, an amplifier and an attenuator are adopted at the output and input ports of the network analyzer for controlling the microwave power irradiated on the ceramic metamaterial sample. The power is measured with a power meter by using convenient relative units named dBm. A value dBm or decibel-milliwatts *P*[dBm] is a level of power in decibels referenced to 1 mW by the definition *P* [dBm] = 10 log_10_(*P*[mW]/1[mW]).

## Data Availability

All data needed to evaluate the conclusions in the paper are present in the paper. Additional data related to this paper may be requested from the authors.
